# The Distribution Characteristics of Vegetation in the Subrange of the Altai Mountains, Xinjiang

**DOI:** 10.3390/plants12223915

**Published:** 2023-11-20

**Authors:** Qiumei Cao, Yan Wei, Wenjun Li, Ying Feng, Ozodbek S. Abduraimov

**Affiliations:** 1 Grassland College, Xinjiang Agricultural University, Urumqi 830011, China; caoqiumei@ms.xjb.ac.cn; 2 CAS Key Laboratory of Biogeography and Bioresource in Arid Land, Xinjiang Institute of Ecology and Geography, Urumqi 830011, China; liwenjunao@ms.xjb.ac.cn (W.L.); luckfy@ms.xjb.ac.cn (Y.F.); 3 The Specimen Museum of Xinjiang Institute of Ecology and Geography, Chinese Academy of Sciences, Urumqi 830011, China; 4 Institute of Botany, Academy Science Republic of Uzbekistan, Tashkent 100126, Uzbekistan; ozodbek88@bk.ru

**Keywords:** Xinjiang Altai subrange, vegetation type, composition and distribution characteristics

## Abstract

The Altai Mountains are an important center of biodiversity and are a major habitat for threatened and endemic species in Asia. Moreover, the Altai Mountains are a valuable site for the study of the evolution of central Asian vegetation. The Xinjiang Altai subrange represents the largest part of the southern Altai Mountains and has many unique plant communities. After conducting a thorough literature review and field investigation, we utilized the Chinese vegetation categorization system to identify the dominant plant communities in the Xinjiang Altai subrange and report their composition and distribution characteristics. Our results show that (1) the natural plant communities present in the Xinjiang Altai subrange can be divided into eight vegetation types, eighteen vegetation subtypes, and 50 communities. Among these, two communities—Form. *Calligonum rubicundum* and Form. *Seriphidium borotalense*-*Festuca valesiaca*—are present only in the Xinjiang Altai subrange. (2) The Xinjiang Altai subrange is located at the junction of three major biomes containing unique vegetation types (coniferous forest, temperate broadleaf forest, and desert). Thus, the Xinjiang Altai subrange is distinct in its staggered transition from mountainous boreal taiga to temperate desert. This research provides textual data to contextualize the cultural heritage of the Xinjiang Altai subrange and also provides a scientific basis for the protection and sustainable management of natural resources found in the Xinjiang Altai.

## 1. Introduction

Vegetation classification is an important part of biogeographical research and also comprises one of the research’s most complex problems [1,2]. Vegetation classification can improve our understanding of the structural characteristics of vegetation, along with the species composition and the relationship between different plant species and their environment [3,4]. Meanwhile, the research results of vegetation classification can provide a scientific basis for the formulation of and measures within laws and regulations related to the protection and rational utilization of vegetation [5,6,7]. 

The Altai (synonym: Altay) Mountain Range spans much of Central Asia and crosses Russia, Kazakhstan, China, and Mongolia. It is the largest mountain range in the Western Siberian biogeographic region and is an important center of biodiversity for many species found in ecosystems throughout northern and central Asia [8,9,10,11]. The Xinjiang Altai Mountains are located on the southern side of the middle portion of the range. This subrange is among the most abundant in plant diversity and vegetation in the whole Altai range and marks the most southerly distribution of many species of Europe–Siberian flora [12,13]. Moreover, the Xinjiang Altai subrange is the most important migration channel for wildlife in the Altai Mountains. In addition to seasonal tourist activities and grazing, there is no interference from industrial projects or agricultural production in the mountains, and the Altai Mountains have preserved a unique natural and original vegetation landscape. From west to east, a series of natural altitudinal zones are present along the southern slope of the Xinjiang Altai subrange. This has created a succession of zonal landscapes from Arctic tundra, alpine meadow, subalpine meadow, Europe–Siberian taiga forest, and the north forest steppe to the Central Asia desert steppe [14,15]. These zonal landscapes are typical of the southeastern Altai Mountains, and they exhibit gradual changes in response to different atmospheric conditions [16]. The geographical distribution of endemic flora and the presence of special vegetation types, as well as an unbroken vertical zone in the Xinjiang Altai subrange, result in this area being ideal for studying many characteristics of different plant species. In addition to its scientific value, the Xinjiang Altai subrange is also important for the conservation of wild species.

However, since the end of the 20th century, the disorderly development taking place over successive years, grassland overgrazing, grassland degradation, local forest overcutting, aging of forests, and the vigorous development of tourism have all caused serious deterioration in the ecological environment of the Xinjiang Altai subrange [17]. Meanwhile, the forest vegetation has degraded, important biological resources have been sharply reduced, and rare species have become endangered [18]. The problem of vegetation restoration and sustainable utilization of resources is a prominent one that places serious restrictions on the sustainable development of society. Research on the vegetation of the Xinjiang Altai subrange has mostly focused on small local areas within the mountain system. Some scholars have explored the relationship between pollen assemblage, vegetation, and the climatic environment on a macroscale [19,20,21] and have studied the response of local vegetation cover and major coniferous tree species to climate change [22,23,24]. So far, there have been no systematic studies of the biogeographical distribution of the vegetation present in the Xinjiang Altai subrange, which is regarded as a natural geographical unit [25,26,27]. Therefore, the characteristics of the vegetation types and the community composition of the Xinjiang Altai subrange were the subject of this research in order to provide a scientific basis for the selection of vegetation restoration species, community construction, protection, and sustainable management of natural resources found in the Xinjiang Altai subrange. At the same time, the third scientific expedition to Xinjiang was launched in 2021, so this research also provided textual data for the third scientific investigation of the Irtysh River basin in Xinjiang.

## 2. Materials and Methods

### 2.1. Study Area

**Figure 1:**
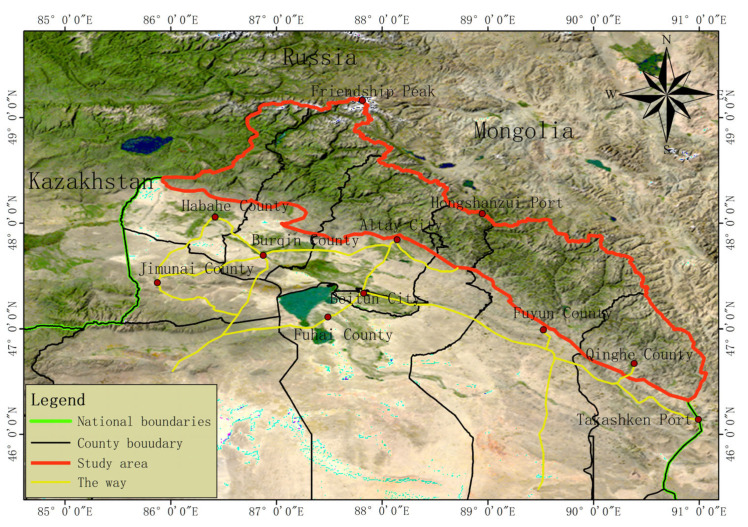
The location of the study area.

The Xinjiang subrange of the Altai Mountains (approximate coordinates: 45–49° N, 86–94° E) is located 750 km long and 60–140 km wide along the northern border of the Xinjiang Uygur Autonomous Region, China. The subrange spans from the China–Kazakhstan border in the west to Mulei County, Xinjiang Province in the east, Russia in the north, and the Gurbantunggut Desert (China) in the south (Figure 1). The average annual temperature is −0.2 °C, and the average annual precipitation is 200–1000 mm. The main soil types in the Xinjiang Altai subrange are brown calcium-rich soil, chestnut soil, mountain grey forest soil, subalpine meadow soil, alpine meadow soil, and mountain tundra soil. The soil type, morphological characteristics, and vertical belt structure of the Xinjiang Altai subrange are typical of mountainous soils and provide a suitable habitat for the occurrence and succession of vegetation in this area and the proliferation of wild plant populations.

### 2.2. Survey Methods

**Figure 2:**
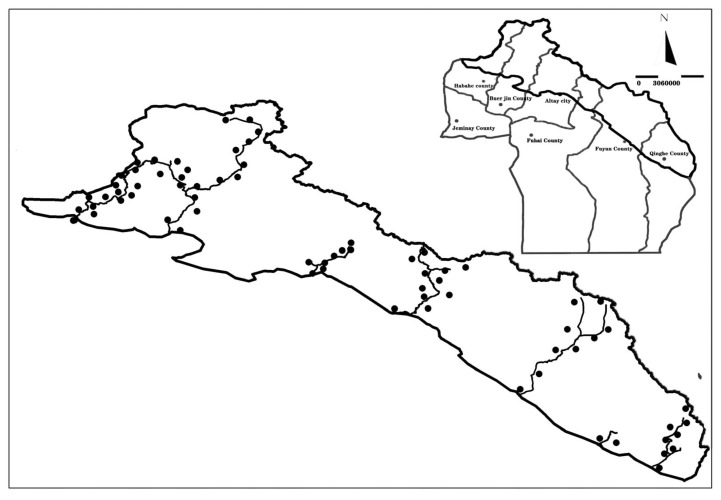
The plot map.

Based on the interpreted remote sensing images of the Altai Mountains and the vegetation map of Xinjiang, seven vertical transects were selected in the survey area from 2012 to 2021 (Figure 2). The typical sample plots were set up in the area with a uniform distribution of dominant species around each sample line [28,29]. The sample area was determined as per the minimal area method of phytocoenology [30]. The area of the sample plot was 100 × 100 m. Five 25 × 25 m arbor plots, five 10 × 10 shrub plots, and five 1 × 1 herb plots were arranged in the four corners and the center of the sample plot. The types and numbers of trees, shrubs, and herbs in the quadrant were counted, and three to five standard plants were selected for each plant. The plants’ height, crown width, and coverage were measured. The longitude, latitude, and altitude were recorded as environmental variables.

The important value (calculated below) was used to evaluate the dominance of the plant population within the community.

The important value of each species was calculated as follows: IV(%) = (Relative height + relative density + relative frequency+ relative coverage)/4.

### 2.3. Vegetation Map Compilation

**Figure 3:**
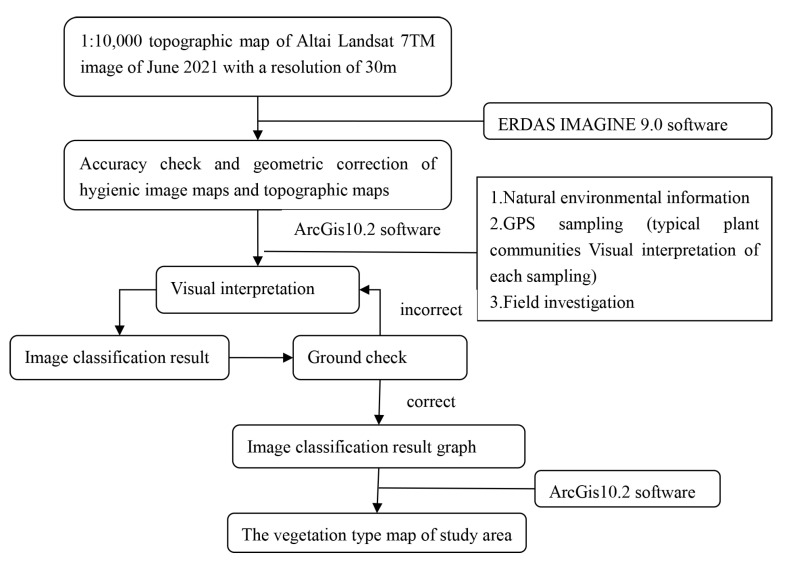
The specific process of Vegetation map compilation.

We drew the vegetation map with 3S technology [31,32]. The specific process is as follows: The vegetation map was based on the topographic map of 1:10,000 in the Aletai region of Xinjiang and the Landsat 7^TM^ image of the Altai region with a resolution of 30 m in June 2021. The topographic map of 1:10,000 in the study area was geometrically corrected with the software ERDAS IMAGINE (version 9.0), and the satellite imagery was corrected using the corrected 1:10,000 topographic map. The vegetation distribution data (including natural environment data, field sample field surveys, GPS sample surveys, and vegetation distribution literature data) were vectorized using the ArcGIS software (version 10.2). The GPS sample, topographic map, and satellite image map were superimposed by ArcGIS software to mark the spot attributes. According to the main basis of ground-landscape type discrimination, the remote sensing satellite imagery was visually interpreted and mapped (Figure 3). Finally, through ground verification, the original image of the visual interpretation was modified several times, and the vegetation map was completed.

### 2.4. Data Processing

Montane vegetation was classified according to species composition as well as the ecological and geographical characteristics of the plant communities. We used the Chinese vegetation classification method, which classifies plant distribution according to a type-subtype-formation/community system [1,33]. For instance, a forest type (e.g., deciduous broadleaf forest) can be divided into multiple subtypes (e.g., mountain and river valley deciduous broadleaf forests). We performed plant dominance assessments to identify the dominant species in each plot type [34]. We compiled survey data to classify the natural vegetation found in the Xinjiang Altai subrange according to this system.

## 3. Results

### 3.1. The Floral Diversity of the Xinjiang Altai Subrange

Our results suggest that the natural vegetation found in the Xinjiang Altai subrange can be divided into eight vegetation types, eighteen vegetation subtypes, and 50 formations or communities (Table 1).

**Figure 4:**
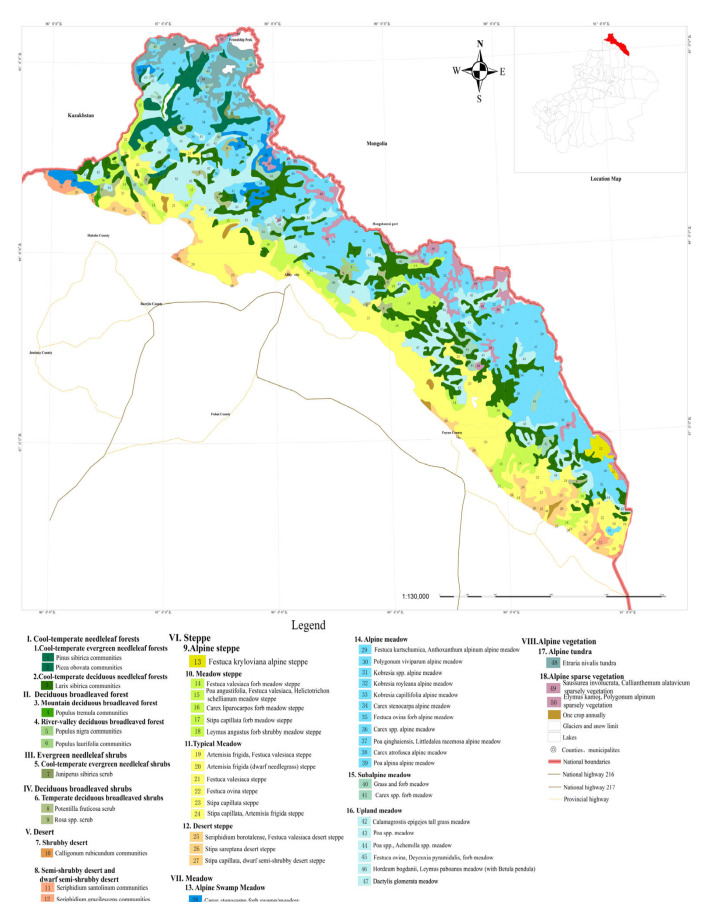
Vegetation map of Xinjiang Altai subrange.

The zonal vegetation of cold-temperate needleleaf forests, mainly distributed on hillsides and river valleys that face wet air currents, accounts for 15.89% of the total area and consists of cold-temperate tree species (Figure 4). Cold-temperate needleleaf forests include two vegetation subtypes: cold-temperate evergreen needleleaf forests and cold-temperate deciduous needleleaf forests. The main species present in cold-temperate needleleaf forests include *Pinus sibirica* Du Tour, *Picea obovata* Ledeb., *Abies sibirica* Ledeb. and *Larix sibirica* Ledeb.

Deciduous broadleaf forests are located in valleys and on the lower parts of the slopes of older mountains in the Xinjiang Altai subrange; these areas account for 0.30% of the total area of the subrange. Deciduous broadleaf forests contain two vegetation subtypes: mountain and river valley deciduous broadleaf forests. The predominant species present in this vegetation type include *Populus tremula* L. and *Populus nigra* L.

The evergreen conifer and deciduous broadleaf shrub vegetation types each included only a single vegetation subtype. These are cold-temperate evergreen coniferous shrubs and temperate deciduous broadleaf shrubs, respectively. Shrub vegetation in the Xinjiang Altai subrange is generally not zonal but is instead widely distributed and accounts for ~0.11% of the total area. The most predominant shrubs present in the Xinjiang Altai subrange include *Juniperus sibirica* Burgsd., *Potentilla fruticosa* (L.) Rydb. and *Cotoneaster* spp.

The desert vegetation type is narrowly distributed in the Xinjiang Altai subrange and is only present along riverside terraces, mountainous alluvial fans, and mountain plains along the Irtysh and Wulungu Rivers at an altitude of 600–800 m. These zones contain shrubby desert, semi-shrubby desert, and dwarf semi-shrubby desert vegetation subtypes.

Steppe vegetation communities in the Xinjiang Altai subrange are zonal and generally consist of perennial herbaceous plants. Steppe vegetation is an important part of the vertical vegetation belt of the Xinjiang Altai subrange, is mainly distributed in the frontal mountain belt at an altitude of 1100–2300 m, and accounts for 30.56% of the total area. Steppe vegetation has four vegetation subtypes and 15 distinct communities.

Meadow vegetation in the Xinjiang Altai subrange is generally not zonally distributed. Meadow vegetation is widely distributed in the mountains, from subalpine to plains areas, which account for 44.24% of the total area surveyed. Four vegetation subtypes were found to be present: alpine swamp meadow vegetation, alpine meadow vegetation, subalpine meadow vegetation, and typical meadow vegetation. These subtypes contain 20 formations, and together they are the most abundant type of vegetation present in the Xinjiang Altai Mountain subrange. Perennial grasses and moss grasses are the most important structural species in meadows.

Alpine vegetation is distributed in the alpine zone at an elevation of 2600–3300 m in screes, alpine rocks, and tundra. This accounts for 6.53% of the total area of the Xinjiang Altai subrange. This vegetation type contains two vegetation subtypes: alpine tundra and alpine sparse vegetation. Alpine vegetation contains various mosses and lichens, as well as a small number of alpine grasses and small shrubs scattered in the alpine tundra. These plant species are typical of the alpine vegetation found throughout Siberia.

### 3.2. Structural Characteristics of the Main Vegetation Communities Found in the Xinjiang Altai Subrange

Affected by climate and habitat environment, the vegetation composition of the Altai Mountains in Xinjiang is dominated by temperate montane plants, and the typical vegetation community structure characteristics are obviously different (Table 2).

## 4. Discussion

Significant differences in topography, local microclimate, hydrothermal conditions, and soil content provide suitable conditions for the development and maintenance of multiple vegetation types in the Xinjiang Altai subrange [35]. This climatic variability is due to the influences of the cold and humid airflows from the Arctic and Atlantic Oceans, the Siberian–Mongolian high-pressure dry anticyclone system, and the variability in landform types [36,37]. The types of vegetation found in the Xinjiang Altai subrange can be divided into eight main types, eighteen subtypes, and 50 formations/communities. Many types of vegetation had obvious zonal characteristics. Cold-temperate coniferous forests constitute the largest part of the total vegetation present; meanwhile, both grassland and meadow vegetation were also widely developed in the Xinjiang Altai subrange. The *Calligonum rubicundum*, *Seriphidium borotalens*, *and Festuca valesiaca* desert steppe communities were present only in the Xinjiang Altai subrange, and they represent a special type of abundant vegetation in the whole Altai Mountains.

The zonal vegetation is cold-temperate coniferous forest, which belongs to the Southern Taiga Forest; the distribution elevation of this vegetation is varied greatly with climatic factors. It is distributed in the northwestern part of the mountain at 1100–2300 m. However, in the southeastern part of the mountain, the distribution altitude has increased, between 1300 and 2600 m, which is influenced by two factors: the low trend of the mountain itself and the arid airflow in Mongolia. From the west to the east of the mountain, the vegetation type not only changes its distribution range but also changes its composition, from *Picea obovata* Ledeb. and *Abies sibirica* Ledeb., to *Larix sibirica* Ledeb., which is the adapted cold and wet climate to the continental climate. The distribution of vegetation communities throughout the Xinjiang Altai subrange is affected by the local microclimate and the arid airflow from Mongolia; here, cold-tolerant mesophytes gradually give way to drought-tolerant mesoxerophytes as the latitude increases [38,39]. This pattern conforms to the patterns of plant community spatial distribution—i.e., from cold to warm- and dry-adapted plants; this is as predicted by community ecology theory [40,41]. The bio-ecological process of plant community type evolution from cold to warm–dry type on the south slope of Altai Mountain was fully demonstrated.

Vertical vegetation zones are an important feature of alpine vegetation distributions [42,43]. As the elevation of the mountain rises, the temperature decreases, the sunshine and wind increase, and precipitation increases. In the Xinjiang Altai subrange, the vegetation shows a complete vertical sequence along the southern slope of the Altai Mountains, with noticeable elevation gradients. From top to bottom, the altitudinal zones are as follows: ice/snow, alpine tundra, alpine sparse vegetation, alpine meadow, subalpine meadow, forest steppe, mountain steppe, and desert steppe. The natural altitudinal zones in the Xinjiang Altai subrange and along the north slope of the Altai Mountains constitute a complete and natural vertical spectrum of the Altai Mountains.

The Altai Mountains are oriented from the northwest to the southeast. The higher the temperature, the smaller the precipitation in the southeast. Therefore, the vegetation vertical belt structure in the Xinjiang Altai Mountains also shows a difference. The main performance is as follows: (1) The base belt of the vertical natural zone of the Kanas area is the forest steppe zone, which is located within the more humid western region of the Xinjiang Altai Mountains. Meanwhile, throughout the southeastern Fuyun and Qinghe areas with relatively little precipitation, the vegetation vertical natural zone is developed on the relatively arid desert steppe zone; (2) The vertical boundary of each zone in the vertical zone of the vegetation rises, the mountain steppe zone expands upwards, and the meadow vegetation zone is narrow. (3) The southern Taiga occupies the largest proportion, which is adapted to the cold and humid climate in the western part of the Altai Mountains; in the middle of the mountain, the steppe and semi-desert vegetation are extensively developed, and the forest is dominated by relatively drought-tolerant deciduous coniferous forest; the mountainous vegetation to the southeast is more afflicted by drought; the forest steppe zone in the southeast has replaced the forest meadow zone in the west; and the subalpine vegetation in the southeast shows different degrees of grassing. Therefore, many scholars have summarized this type of vertical belt structure as the vertical belt structure-type group of the Siberian–Mongolian mountain vegetation [44].

The distribution of vegetation is not only affected by environmental factors, which are dominated by heat and moisture, but is also related to plants’ individual adaptability and variability to the environment and its location and geological history [45]. Therefore, due to the heterogeneity of the environment in different regions, there are also obvious differences in the flora. Due to the complex and ever-changing geological features of the mountainous areas, the altitude changes greatly and the climate is divided, resulting in a large difference in the flora of each mountain system.

In this large-scale pattern, the Altai Mountains developed a variety of vegetation types. The Russian Altai Mountains, located on the northern slopes of the Altai Mountains, are the distribution center of the dark Taiga vegetation type, which is the typical Siberian jungle consisting of *Abies sibirica* Ledeb., *Cedrus deodara* (Roxb.) G. Don, and *Populus davidiana* Dode et al. [29]. Due to the influence of the Atlantic airstream, the area is home to a large number of sub-alpine meadows and alpine meadows that are rare in other Siberian mountain regions. The flora in the area is dominated by elements of Siberian flora. The Altai Mountains of Mongolia are located on the western edge of the Mongolian Plateau and on the southeast slope of the Altai Mountains. Due to the mountains that surround the Mongolian Plateau, the entry of humid airflow is blocked. Therefore, due to the local Mongolian high pressure, the main vegetation types on this slope are desert and desert grasslands [46]. The low slopes and valleys of the Altai Mountains of Mongolia are dotted with mountainous sub-desert belts. Due to the combined effects of geographical location and climate, the forest belt has disappeared in the Altai Mountains of Mongolia and is only scattered in the valley. The slope is dominated by drought-tolerant ancient Mediterranean components. The Kazakh Altai subrange in the southwestern slope of the Altai Mountains is located in the European–Central Asian grassland area, which has developed a wide range of temperate broad-leaved deciduous forests and typical grassland vegetation types [47]. The forest and grassland vegetation types on this slope are mainly *Pinus sylvestris* L., *Populus tremula* Linn., *Betula pendula* Roth., *Stipa* spp., *Koeleria* spp., *Festuca* spp., etc. The forest steppe is dotted with more wetland marshes. Plants are mainly comprised of species in the frigid zone, subfrigid zone, and grassland area. In the southeastern mountain belt and plains of the Kazakhstan Altai Mountains, a large number of mesophytes and mesoxerophytes desert plants are distributed, and some ephemeral plants have been added. In the middle of the southern slope of the Altai Mountains, the Xinjiang Altai subrange is at an intersection zone of three major vegetation types: taiga forest, temperate broadleaf forest, and desert. Due to the control of the arid continental climate of central Asia and the Siberian–Mongolian anticyclone, the slope has provided a good foundation for the occurrence and development of various vegetation types. Due to the close proximity of Russia to the northwest, the climate is relatively humid; in the Xinjiang Altai subrange, the taiga-type coniferous forest of the northern coniferous forest belt enters Xinjiang and forces hard limits on the distribution area of the Eurasian grassland. It is entirely absent, only in a narrow band at the south of the subrange [46,48].

As the Xinjiang Altai Mountains are located at the intersection of the Siberian Circum–North Taiga, Holarctic coniferous forest, Eurasian mountain broad-leaved forest, Central Asian grassland belt, and Mongolian desert basin, the vegetation of this slope is not as single as the dark taiga of Russia’s Altai Mountains, unlike the Altai Mountains in Kazakhstan, which dominate most of the mountains by the real steppe. Unlike the Mongolian Altai Mountains, which have disappeared from the forest belt. This special geographical location makes it possible for the Xinjiang Altai subrange to host a wide variety of plant communities. It has obvious features of interlacing and transitioning the cold temperate zone of the taiga forest in the mountainous area and the middle temperate desert zone, thereby causing a unique and irreplaceable change in the spatial distribution pattern of vegetation in the Altai Mountains.

Throughout the Altai Mountains, the extension of the mountain results in each slope being at a different latitude, which, coupled with the diverse geological and geomorphological forms of the mountain and the high difference between precipitation and local photothermal conditions of the mountain, has led to the diversification of vegetation types in the region. The vegetation from the south to the north is transformed from the humidity- and shade-tolerant dark taiga vegetation type to the mesophytes and mesoxerophytes of the steppes and desert steppes; the vegetation from west to east is changed from the dark taiga forest to the typical meadow and then temperate broadleaf forest in the Altai Mountains of Xinjiang, and finally turned into the Gobi grassland in the Altai Mountains of Mongolia.

## 5. Conclusions

In our study, we identified eight vegetation types, 18 vegetation subtypes, and 50 formations, demonstrating a diverse range of vegetation types within the Xinjiang Altai subrange. Among them is the Form. *Calligonum rubicundum* and Form. *Seriphidium borotalense. Festuca valesiaca* is present only in the Xinjiang Altai subrange, which is unique among the rich vegetation types in the Altai Mountains. The Xinjiang Altai subrange is located at the junction of three major biomes containing unique vegetation types (coniferous forests, temperate broadleaf forests, and deserts). The Xinjiang Altai subrange is distinct in its staggered transition from mountainous boreal taiga to temperate desert. Therefore, it is both unique and irreplaceable in the spatial distribution pattern of vegetation in the Altai Mountains.

## Figures and Tables

**Table 1:** The vegetation types of Altai Mountain, Xinjiang.

Vegetation Type	Vegetation Subtype	Formation/Community	Proportion of Total Area (%)
I. Cold-temperate needleleaf forests	1. Cool-temperate evergreen needleleaf forests	(1) *Pinus sibirica* communities	15.89
		(2) *Picea obovata* communities	
	2. Cool-temperate deciduous needleleaf forests	(3) *Larix sibirica* communities	
II. Deciduous broadleaved forest	3. Mountain deciduous broadleaved forest	(4) *Populus tremula* communities	0.30
	4. River-valley deciduous broadleaved forest	(5) *Populus nigra* communities	
		(6) *Populus laurifolia* communities	
III. Evergreen needleleaf shrubs	5. Cool-temperate evergreen needleleaf shrubs	(7) *Juniperus sibirica* scrub	0.11
IV. Deciduous broadleaved shrubs	6. Temperate deciduous broadleaved shrubs	(8) *Potentilla fruticosa* scrub	1.15
		(9) *Rosa* spp. scrub	
V. Desert	7. Shrubby desert	(10) *Calligonum rubicundum* communities	0.81
	8. Semi-shrubby desert and dwarf semi-shrubby desert	(11) *Seriphidium santolinum* communities	
		(12) *Seriphidium gracilescens* communities	
VI. Steppe	6. Alpine steppe	(13) *Festuca kryloviana* alpine steppe	30.56
	10. Meadow steppe	(14) *Festuca valesiacavarii* forb meadow steppe	
		(15) *Poa angustifolia*, *Festuca valesiaca*, *Helictotrichchon schellianum* meadow steppe	
		(16) *Carex liparocarpos* forb meadow steppe	
		(17) *Stipa capillata* forb meadow steppe	
		(18) *Leymus angustus* forb shrubby meadow steppe	
	11. Typical Meadow	(19) *Artemisia frigida*, *Festuca valesiaca* steppe	
		(20) *Artemisia frigida* (dwarf needlegrass) steppe	
		(21) *Festuca valesiaca* steppe	
		(22) *Festuca ovina* steppe	
		(23) *Stipa capillata* steppe	
		(24) *Stipa capillata*, *Artemisia* frigida steppe	
	12. Desert steppe	(25) *Seriphidium borotalense*, *Festuca valesiaca* desert steppe	
		(26) *Stipa sareptana* desert steppe	
		(27) *Stipa capillata* dwarf semi-shrubby desert steppe	
VII. Meadow	13. Alpine Swamp Meadow	(28) *Carex stenocarpa* forb swamp/meadow	44.24
	14. Alpine meadow	(29) *Festuca kurtschumica*, *Anthoxanthum alpinum* alpine meadow	
		(30) *Polygonum viviparum* alpine meadow	
		(31) *Kobresia* spp. alpine meadow	
		(32) *Kobresia royleana* alpine meadow	
		(33) *Kobresia capillifolia* alpine meadow	
		(34) *Carex stenocarpa* alpine meadow	
		(35) *Festuca ovina* forb alpine meadow	
		(36) *Carex* spp. alpine meadow	
		(37) *Poa qinghaiensis*, *Littledalea racemosa* alpine meadow	
		(38) *Carex atrofusca* alpine meadow	
		(39) *Poa alpina* alpine meadow	
	15. Subalpine meadow	(40) *Festuca* spp., forb meadow	
		(41) *Carex* spp., forb meadow	
	16. Upland meadow	(42) *Calamagrostis epigeios* tall grass meadow	
		(43) *Stipa* spp. meadow	
		(44) *Stipa* spp., *Achemilla* spp. meadow	
		(45) *Festuca ovina*, *Deyeuxia pyramidalis* forb meadow	
		(46) *Hordeum bogdanii*, *Leymus paboanus* meadow (with *Betula pendula*)	
		(47) *Dacylis glomerata* meadow	
VIII. Alpine vegetation	17. Alpine tundra	(48) *Etraria nivalis* tundra	6.53
	18. Alpine sparse vegetation	(49) *Saussurea involucrata*, *Callianthemum alatavicum* sparsely vegetated community	
		(50) *Elymus kamoji*, *Polygonum alpinum* sparsely vegetated community	

**Table 2:** Structural characteristics of the typical vegetation communities in the Xinjiang Altai subrange.

Formation/Community	Distribution Regions	Altitude (m)	Soil Type	Distribution Area	Main Representative	Population Density (%)	Community Height (m)	Canopy Closure
(1) *Pinus sibirica* communities	In the upper reaches of Kanas Lake and the Homer River	1600–1900	Mountain gray forest soil	0.58	Tree layers: *Pinus sibirica* Du Tour, *Picea obovata* Ledeb., *Larix sibirica* Ledeb.; shrub layer: *Lonicera caerulea* var. *altaica* Pall., *Juniperus sibirica* Burgsd., *Vaccinium myrtillus* L; herbaceous layer: *Caltha palustris* L., *Pyrola* spp., *Moneses uniflora* (Linn.) A. Gray, and *Luzula pallescens* (Whlb.) Sw.; the moss layer: was widely developed, covering more than 25% of the area, and the main moss present was *Dicranum spurium* Hedw.	70	15.00–25.00	0.70–0.80
(2) *Picea obovata* communities	Distributed along low-elevation valley margins or in the moist valley terraces of the forest steppe	1300–2100	Peat mire or dark gray forest soil	2.37	Tree layers: *Picea obovata* Ledeb., *Larix sibirica* Ledeb., *Betula pendula* Roth.; shrub layer: *Rosa acicularis* Lindl. RoL Monr., *Ribes nigrum* L.; herbaceous layer: *Pyrola* spp., *Corallorhiza trifida* Chatel, and *Adoxa moschatellina* L.; the moss layer: were widespread, covering 75–85% of the area. *Brachythecium albicans* (Hedw.) B. S. G. and *Hylocomium splendens* (Hedw.) B. S. G., were the most common.	80	14.00–20.00	0.6
(3) *Larix sibirica* communities	Widely distributed on the shaded and half-shaded slopes along the central and southeastern parts of the Xinjiang Altai subrange	west: 1300–2300; southeastern: 1700–2600 m	Mountain gray forest soil, and turfgrass predominated the soil was highly strengthened	13.01	Tree layers: *Larix sibirica* Ledeb., *Picea obovata* Ledeb., *Pinus sibirica* Du Tour, *Betula pendula* Roth., *Populus tremula* L., *Sorbus sibirica* Hedl., shrub layer: *Cotoneaster* spp., *Lonicera* spp., *Spiraea* spp., *Betula rotundifolia* Spach; Herbaceous layer: *Kobresia* spp., *Dactylis glomerata* L., *Deyeuxia pyramidalis* (Host) Veldkamp, *Eragrostis pilosa* (L.) Beauv.; The distribution of mosses in the forest was limited.	80	15.00–17.00	0.70–0.90
(4) *Populus tremula* communities	On the slopes of valleys and lakeside terraces or the edge of patches of the *Larix sibirica* formation.	1500	Brown coniferous soil	0.07	Tree layers: *Populus tremula* L., *Betula pendula* Roth., *Larix sibirica* Ledeb., *Picea obovata* Ledeb.; shrub layer: *Cotoneaster* spp., *Spiraea* spp., *Lonicera* caerulea var. altaica Pall.; Herbaceous layer: *Calamagrostis epigeios* (Linn.) Roth, *Elymus repens* Desv., *Dactylis glomerata* L., *Geranium pseudosibiricum* J.; Moss was occasionally also seen in this formation.	60	15.00–25.00	0.30–0.50
(5) *Populus nigra* communities	Along the banks of the Irtysh River in the south of the Altai Mountains	600	Brown forest soil	0.10	Tree layers: *Populus nigra* L., *Populus alba*, *Populus* × *jrtyschensis*, *Populus alba* L., shrub layer: *Rosa acicularis* Lindl. and different willow shrubs (*Salix pentandra* L., Salix triandra L., Salix turanica Nas.); Herbaceous layer: Poa nemoralis L., *Aquilegia glandulosa* Fisch. ex Link., *Geum aleppicum* Jacq.	60–70	10.00–15.00	0.2
(6) *Populus laurifolia* communities	Low-altitude plains in the Xinjiang Altai subrange	600–900	Brown calcium-rich soil	0.14	Tree layers: *Populus laurifolia* Ledeb., *Populus alba* L., *Betula pendula* Roth.; shrub layer: *Rosa acicularis* Lindl., *Ribes pulchellum* Turcz., *Cotoneaster oliganthus* Pojark.; Herbaceous layer: *Phragmites australis* (Cav.) Trin., *Juncus* spp., *Elytrigia repens* (L.) Nevski	60–75	10.00–12.00	0.20–0.40
(7) *Juniperus sibirica* scrub	In the middle of shady slopes	1400–1600	Rocky shrubby-meadow soil	0.11	Shrub layer: *Juniperus communis* var. *saxatilis* Pall., *Cotoneaster uniflorus* Bge., *Lonicera hispida* Pall. ex Roem. et Schult, *Rosa acicularis* Lindl.; Herbaceous layer: *Iris ruthenica* Ker-Gawl., *Phlomis oreophila* Kar., *Poa pratensis* L.	50–60	0.50–1.00	
(8) *Dasiphora fruticosa* scrub	On sunny slopes southwest of the Altai Mountains	2400–2600	Alpine shrub meadow soil	0.21	Shrub layer: *Dasiphora fruticosa* (L.) Rydb., *Salix sclerophylla* Anderss., *Spiraea alpina* Pall., *Dasiphora parvifolia* (Fisch. ex Lehm.) Juz.; Herbaceous layer: *Stipa purpurea* Griseb., *Dracocephalum heterophyllum* Benth., *Carex atrofusca* Schkuhr., etc.	50–65	0.50–1.00	
(9) *Rosa* spp. scrub	On the northern slope of mountains	1300–1800	Mountain chernozem	0.94	This community type is also one of the most typical communities in temperate deciduous shrubland. shrub layer: *Rosa spinosissima* Linn., *Rosa acicularis* Lindl., *Rosa platyacantha* Schrenk, *Rosa laxa* Retz., *Spiraea hypericifolia* L., *Cotoneaster melanocarpus* Lodd. and various *Ribes* spp.; Herbaceous layer: Calamagrostis epigeios (Linn.) Roth, *Geum aleppicum* Jacq., *Achillea millefolium* Linn.	70	1.50–2.00	
(10) *Calligonum rubicundum* communities	Narrowly distributed along the south bank of the Irtysh River	600	Sand or gravel soil	0.03	This formation is usually constituted a sparse mono-dominant community of *Calligonum rubicundum* Bunge, and it included few companion species. shrub layer: *Astragalus gebleri* Fisch. ex Bong., *Atraphaxis frutescens* (L.) Eversm., *Kochia prostrata* (L.) Schrad.; Herbaceous layer: *Corispermum lehmannianum* Bunge, *Stipa caucasica* subsp. glareosa (P. A. Smirn.) Tzvelev	>10%	1.00–1.50	
(11) *Seriphidium santolinum* communities	In sandy areas in the riparian terrace of the Irtysh and Ulungur rivers	600–700	Sandy soil	0.05	The species composition of the community was simple and often included many grasses. Herbaceous layer: *Seriphidium santolinum* (Schrenk) Poljak., *Bassia prostrata* (L.) Beck, *Corispermum lehmannianum* Bunge, *Eremopyrum triticeum* (Gaertn.) Nevski	20–30	0.20–0.30	
(12) *Seriphidium gracilescens* communities	It was rare and found only in mountain alluvial fan or mountain plain areas in mountain alluvial fan or mountain plain areas	600–900	Gravel and calcareous soil	0.72	The community structure was relatively simple. Herbaceous layer: *Seriphidium gracilescens* (Krasch. et Iljin) Poljak., *Nanophyton erinaceum* (Pall.) Bge.), *Artemisia frigida* Willd., *Atraphaxis decipiens* Jaub. et Spach	10–18	0.15–0.30	
(13) *Festuca kryloviana* alpine steppe	In the subalpine zone in the eastern central Altai Mountains	2200–2600	Subalpine meadow soil	0.57	Herbaceous layer: *Festuca kryloviana* Reverd., *Draba nemorosa* L., *Bistorta officinalis* Raf., *Festuca kurtschumica* E. Alexeev	85	0.20–0.25	
(14) *Festuca valesiacavarii* forb meadow steppe	Distributed below the forest belt	1200–1800	Mountain chestnut soil	3.08	This community type was typical of mountain meadow grasslands found in the Xinjiang Altai subrange. Herbaceous layer: *Festuca valesiaca* subsp. *sulcata* (Hackel) Schinz et R. Keller, *Heteropappus altaicus* (Willd.) Novopokr., *Dracocephalum nutans* Linn. and *Gagea fedtschenkoana* Pasch.	50	0.20–0.50	
(15) *Poa angustifolia, Festuca valesiaca, Helictotrichchon schellianum* meadow steppe	In the frontal mountain belt and mid-mountain zone of the Xinjiang Altai subrange	1200–1800	Chestnut soil and chernozem	1.18	Herbaceous layer: *Poa pratensis* subsp. *angustifolia* (L.) Lejeun., *Festuca valesiaca* subsp. *sulcata* (Hackel) Schinz et R. Keller, *Avenula pubescens* (Huds.) Dumort., *Phleum phleoides* (L.) Karst., *Geum rivale* L., *Thalictrum minus* L.	60–70	0.20–0.60	
(16) *Carex liparocarpos* forb meadow steppe	On sunny and half sunny slopes	1200–1800	Chestnut soil	2.00	Herbaceous layer: *Carex liparocarpos* Gaudin, *Myosotis suaveolens* Waldst. et Kit., *Campanula albertii* Trautv., *Aster altaicus* Willd.	40–55	0.15–0.30	
(17) *Stipa capillata* forb meadow steppe	In mountain valleys	1400–1800	Mountain chernozem soil	0.32	This community type was dominant in the mountainous steppe of the Xinjiang Altai subrange. Herbaceous layer: *Stipa capillata* L., *Alopecurus pratensis* L., *Phlomoides oreophila* (Kar. & Kir.) Adylov, Kamelin & Makhm., *Pulsatilla patens* (L.) Mill., *Anemone sylvestris* L.	50–70	0.30–0.50	
(18) *Leymus angustus* forb shrubby meadow steppe	On rocky and sunny mountain slopes near coniferous forests and grassland shrubs	1400–2000	Rocky mountain chernozem soil	0.52	This community was often present as invasive shrubs, and the plant cover of the shrub layer ranged from 15 to 20%. shrub layer: *Spiraea hypericifolia* L., *Cotoneaster uniflorus* Bge., *Rosa spinosissima* L.; Herbaceous layer: *Leymus angustus* (Trin.) Pilger, *Phleum pratense* L., *Poa pratensis* subsp. *angustifolia* (Linnaeus Lejeun, *Helictotrichon hookeri* (Scribn.), *Galium spurium* L., *Androsace septentrionalis* L., *Achillea millefolium* L.	40–60	0.15–0.20	
(19) *Artemisia frigida, Festuca valesiaca* steppe	In the mid-mountain zones	1400–1700	Mountain chestnut soil	0.41	Shrub layer: *Spiraea hypericifolia* L., *Lonicera caerulea* L., *Rosa spinosissima* L.; Herbaceous layer: *Artemisia frigida* Willd., *Festuca valesiaca* Schleich. ex Gaudin, *Koeleria* spp., *Stipa capillata* L., *Phleum pratense* L.	25–35	0.30–0.60	
(20) *Artemisia frigida* (dwarf needlegrass) steppe	In the Altai steppe zone	1300–2000	Mountain chestnut soil		Herbaceous layer: *Artemisia frigida* Willd., *Carex liparocarpos* Gaudin, *Oxytropis* spp. and *Galium verum* L.	30	0.20–0.30	
(21) *Festuca valesiaca* steppe	The most widely distributed type of steppe in the Xinjiang Altai subrange, appeared on sunny types	1100–1300	Mountain chestnut soil	13.60	Herbaceous layer: *Festuca valesiaca* Schleich ex Gaud, *Carex* spp., *Artemisia frigida* Willd., *Oxytropis* spp., *Scorzonera* spp., *Leontopodium* spp.	50–60	0.10–0.30	
(22) *Festuca ovina* steppe	In the forest-steppes	1200–1800	Brown calcium-rich soil	1.45	Herbaceous layer: *Festuca ovina* L., *Artemisia frigida* Willd., *Psathyrostachys juncea* (Fisch.) Nevski, *Sedum hybridum* L., *Astragalus* spp., *Patrinia intermedia* (Horn.) Roem.	50–65	0.15–0.25	
(23) *Stipa capillata* steppe	The most common mountainous steppe in the Altai mountains, and is found on all (shady, semi-shady, and sunny) slopes in the mid-mountain zone	600–1100	Mountain chestnut soil	2.75	Shrub layer: *Spiraea hypericifolia* L.; Herbaceous layer: *Stipa capillata* L., *Artemisia frigida* Willd., *Carex liparocarpos* Gaudin, *Agropyron cristatum* P. Beauv.	60	0.20–0.40	
(24) *Stipa capillata, Artemisia* frigida steppe	In shady and semi-shady slopes in the Xinjiang Altai subrange	1500–1700	Mountain chestnut soil	1.21	Herbaceous layer: *Stipa capillata* L., *Artemisia frigida* Willd., *Agropyron cristatum* P. Beauv., *Ephedra intermedia* Schrenk, *Elymus kamoji* (Ohwi) S. L. Chen.	50–60	0.10–0.30	
(25) *Seriphidium borotalense, Festuca valesiaca* desert steppe	In the mountainous alluvial fan of the western end of the Xinjiang Altai subrange	1000–1500	Brown calcium-rich soil	0.73	In this community type, *Seriphidium borotalense* was the structural species and *Festuca valesiaca* was the main companion species. Herbaceous layer: *Koeleria* spp., *Atraphaxis frutescens* (L.) Eversm., *Astragalus arbuscula* Pall.	30	0.10–0.30	
(26) *Stipa sareptana* desert steppe	On hillsides and south-facing mountain fronts in the south of the Altai Mountains	1000–1300	Brown calcium-rich soil	2.26	Herbaceous layer: *Stipa sareptana* Becher, *Koeleria* spp., *Leymus ovatus* (Trin.) Tzvel., *Allium polyrhizum* Turcz. ex Regel.	30–50	0.10–0.35	
(27) *Stipa capillata* dwarf semi-shrubby desert steppe	In the lower mountain steppe in the southern Xinjiang Altai subrange	1000–1300	Brown calcium-rich soil	0.23	The structural layer of these communities was chiefly needlegrass, and the companion species included many small shrubs. shrub layer: *Caragana leucophloea* Pojark., *Nanophyton erinaceum* (Pall.) Bunge, Herbaceous layer: *Stipa capillata* L., *Stipa sareptana* Becher	35–45	0.40–0.60	
(28) *Carex stenocarpa* forb swamp/meadow	along shady slopes or glacier-formed valleys in the northwestern Xinjiang Altai subrange.	1500–2000	Deep alpine meadow soil	2.01	Herbaceous layer: *Carex stenocarpa* Turcz. ex V. Krecz., *Poa pratensis* subsp. *angustifolia* (L.) Lejeun., *Geranium rectum* Trautv., *Carex atrofusca* subsp. *minor* (Boott) T. Koyama, *Gentiana algida* Pall., *Phleum phleoides* (Linn.) Karst.	55–65	0.2–0.6	
(29) *Festuca kurtschumica, Anthoxanthum alpinum* alpine meadow	On gentle hillsides or watersheds of alpine zones in the southern Xinjiang Altai subrange	2400–2600	Hypertrophic alpine meadow soils	3.68	Herbaceous layer: *Festuca kurtschumica* E. Alexeev, *Anthoxanthum alpinum* á. Love et D. Love, *Ranunculus altaicus* Laxm., *Viola altaica* Ker-Gawl., *Silene graminifolia* Otth, *Phleum phleoides* (Linn.) Karst.	60–85	0.10–0.30	
(30) *Polygonum viviparum* alpine meadow	In glacial valleys and valley lowlands in the western Xinjiang Altai subrange	2400–2600	Deep alpine meadow soil	0.72	Herbaceous layer: *Polygonum viviparum* L., *Oreomecon nudicaulis* (L.) Banfi, Bartolucci, J.-M. Tison & Galasso, *Anemone sylvestris* L., *Phlomis pratensis* Kar. et Kir., *Phlomoides pratensis* (Kar. & Kir.) Adylov, Kamelin & Makhm., *Carex atrofusca* subsp. *minor* (Boott) T. Koyama	80%	0.10–0.30	
(31) *Kobresia* spp. alpine meadow	Only in the alpine belt in the southeast Xinjiang Altai subrange.	2500	Alpine meadow soil	4.09	Herbaceous layer: *Kobresia myosuroides* (Villars) Foiri, *Kobresia royleana* (Nees) Bocklr., *Anthoxanthum odoratum* L., *Androsace filiformis* Retz., *Carex onoei* Franch. et Savat., *Potentilla chinensis* Ser.	50–65%	0.10–0.20	
(32) *Kobresia royleana* alpine meadow	In alpine meadow soil on shady slopes in the alpine and subalpine belts in the central portion of the Xinjiang Altai subrange	2400–27,000	Alpine meadow soil	4.11	Herbaceous layer: *Kobresia royleana* (Nees) Bocklr., *Myosotis alpestris* F. W. Schmidt, *Primula nivalis* Pall., *Phleum phleoides* (Linn.) Karst.	60–75	0.10–0.15	
(33) *Kobresia capillifolia* alpine meadow	Only in a small area in the northwest corner of the alpine zone of the Xinjiang Altai subrange	2600	Alpine meadow soil	0.09	Herbaceous layer: *Kobresia capillifolia* (Decne.) C. B. Clarke, *Cardamine parviflora* L., *Carex stenocarpa* Turcz. ex V. Krecz., *Saussurea alpina* (Linn.) DC.	60–75	0.10–0.15	
(34) *Carex stenocarpa* alpine meadow	In shady slopes and glacial valleys in the northern and southeastern Xinjiang Altai subrange.	2300–2600	Alpine meadow soil	5.99	Herbaceous layer: *Carex stenocarpa* Turcz. ex V. Krecz., *Bistorta vivipara* (L.) Gray, *Anthoxanthum odoratum* Linn., Saxifraga *hirculus* L., *Koenigia alpina* (All.) T. M. Schust. & Reveal, *Carex rhynchophysa* Fisch., C.A.Mey. & Avé-Lall.	55–70	0.10–0.25	
(35) *Festuca ovina* forb alpine meadow	In alpine and sub-alpine zones in the middle and south-central Xinjiang Altai subrange	2100–2700	Alpine meadow soil	2.37	Herbaceous layer: *Festuca ovina* L., *Festuca rubra* L., *Festuca kurtschumica* E. Alexeev, *Draba alpina* L., *Gentiana algida* Pall.	40–50	0.10–0.30	
(36) *Carex* spp. alpine meadow	Formed patches in the wet shady slopes or U-shaped valleys	2400–2600	Alpine meadow soil	2.44	Herbaceous layer: *Carex atrofusca* subsp. *minor* (Boott) T. Koyama, *Carex dichroa* Freyn, *Carex turkestanica* Rgl., *Carex stenocarpa* Turcz. ex V. Krecz., *Rhodiola quadrifida* (Pall.) Fisch. et Mey., *Ranunculus altaicus* Laxm.	50–65	0.10–0.20	
(37) *Poa qinghaiensis, Littledalea racemosa* alpine meadow	In alpine gullies and elsewhere in alpine and sub-alpine zones in the north and southeastern parts of the Xinjiang Altai subrange	2400–2800	Alpine meadow soil	3.21	Herbaceous layer: *Poa qinghaiensis* Soreng et G. Zhu, *Littledalea racemosa* Keng, *Bistorta vivipara* (L.) Gray, Erigeron eriocalyx (Ledeb.) Vierh., *Carex atrofusca* subsp. *minor* (Boott) T. Koyama	45–60	0.15–0.30	
(38) *Carex atrofusca* alpine meadow	In the shady slopes or valleys in the alpina and sub-alpine zones of the Xinjiang Altai subrange	2500–2800	Alpine meadow soil	2.54	Herbaceous layer: *Carex atrofusca* subsp. *minor* (Boott) T. Koyama, *Cerastium* spp., *Geranium albiflorum* Ledeb., *Pedicularis elata* Willd.	60–70	0.1–0.2	
(39) *Poa alpina* alpine meadow	On sunny slopes or watersheds	2400–2600	Alpine meadow soil	1.55	Herbaceous layer: *Poa alpina* L., *Kobresia bellardii* All., *Elytrigia repens* Desv., *Poa sibirica* Trin.	40	0.1–0.3	
(40) Grass and forb meadow	In the northwestern part of the Xinjiang Altai subrange in gentle watersheds or hillsides	1800–2400	Subalpine meadow soil	0.46	Herbaceous layer: *Anthoxanthum odoratum* L., *Poa alpina* L., *Festuca rubra* L., *Campanula albertii* Trautv., *Gentiana karelinii* Griseb., *Erigeron eriocalyx* (Ledeb.) Vierh.,	60–90	0.1–0.3	
(41) *Carex* spp., forb meadow	Along the slopes of hills and mountains in the Xinjiang Altai subrange	2200–2400	Subalpine meadow soil	1.94	Herbaceous layer: *Carex dichroa* Freyn, *Carex supina* Willd. ex Wahlenb., *Poa altaica* Trin., *Trisetum sibiricum* Rupr., *Festuca rubra* Linn., *Primula algida* Adam, *Ranunculus altaicus* Laxm., *Viola biflora* L.	60–80	0.1–0.4	
(42) *Calamagrostis epigeios* tall grass meadow	Mainly located in the floodplain and inter-valley regions in the northwest and southeast of the Xinjiang Altai subrange	1600–1900	Mountain chernozem	1.42	Herbaceous layer: *Calamagrostis epigeios* (Linn.) Roth, *Trifolium lupinaster* L., *Leymus* spp., *Elymus* spp.	65–75	0.4–1.2	
(43) *Poa* spp. meadow	Widely distributed throughout the mid-mountain zones of the Xinjiang Altai subrange.	1200–2100	Mountain chernozem	10.31	Herbaceous layer: *Sanguisorba alpina* Bge., *Poa sibirica* Trin., *Poa nemoralis* L., *Poa pratensis* subsp. *angustifolia* (L.) Lejeun., *Koenigia alpina* (All.) T. M. Schust. & Reveal, *Sanguisorba alpina* Bge., *Phlomoides pratensis* (Kar. & Kir.) Adylov, Kamelin & Makhm.	70–80	0.3–0.4	
(44) *Poa* spp., *Achemilla* spp. meadow	Along shady slopes in the Liangheyuan Nature Reserve	1700–2300	Mountain chernozem	0.54	Herbaceous layer: *Poa glauca* subsp. *altaica* (Trin.) Olonova et G. Zhu, *Anthoxanthum alpinum* á. Love et D. Love, *Festuca rubra* Linn., *Carex atrofusca* subsp. *minor* (Boott) T. Koyama, Carex onoei Franch. et Savat., *Carex obtusata* Liljebl., *Alchemilla sibirica* Zam., *Alchemilla pinguis* Juz., *Thalictrum minus* Linn.	70	0.3–0.5	
(45) *Festuca ovina, Deyeuxia pyramidalis* forb meadow	Only in the mountain depressions near Lake Kanas	1200–1600	Mountain chernozem	0.08	Herbaceous layer: *Festuca ovina* L., *Deyeuxia pyramidalis* (Host) Veldkamp, *Bistorta vivipara* (L.) Gray, *Carex duriuscula* subsp. *rigescens* (Franch.) S. Y. Liang et Y. C. Tang, *Festuca rubra* L.	55–65	0.3–0.5	
(46) *Hordeum bogdanii*, *Leymus paboanus* meadow (with *Betula pendula*)	Only two areas: in a small area in the low valley in the Qinghe area in the eastern part of the Xinjiang Altai subrange, and in the valley of the Qingli River	1200–1400	Mountain chernozem	0.17	Shrub layer: *Betula pendula* Roth., *Salix pentandra* L., Salix *triandra* L., Herbaceous layer: *Elymus* spp., *Leymus paboanus* (Claus) Pilger, *Calamagrostis epigeios* (Linn.) Roth, *Elytrigia repens* Desv.	80–90	0.6–0.9	
(47) *Dacylis glomerata* meadow	In mountainous areas between Fuhai and Fuyun Counties in the mid-west Xinjiang Altai subrange	1500–1700	Fertile mountain meadow soil	0.80	Herbaceous layer: *Dactylis glomerata* L., *Bromus inermis* Leyss, *Geranium pratense* L., *Sanguisorba alpina* Bunge	80–90	0.6–1.3	
(48) *Flavocetraria nivalis* tundra	Located on gravel hillsides in the Kanas region of the northwestern Xinjiang Altai subrange.	2600	Alpine ice marsh soil	3.06	Herbaceous layer: *Flavocetraria nivalis* (L.) Kärnefelt & A. Thell, *Flavocetraria cucullata* (Bellardi) Kärnefelt & A., *Parmelia* spp., *Potentilla nivea* L., *Waldheimia tridactylites* Kar. et Kir, *Gentiana algida* Pall.	5–10	0.05–0.1	
(49) *Saussurea involucrata*, *Callianthemum alatavicum* sparsely vegetated community	In alpine rock slopes	3000	Alpine ice marsh soil	3.41	Herbaceous layer: *Echeveria laui* Moran & J. Meyrán, *Callianthemum coriandrifolium* Rchb., *Cortusa brotheri* Pax. ex Lipsky, *Pedicularis* spp., *Poa* spp.	<5	0.1–0.3	
(50) *Elymus kamoji*, *Polygonum alpinum* sparsely vegetated community	Only in a small area in the rocky northwest corner of the Kanas region	3200	Alpine ice marsh soil	0.07	Herbaceous layer: *Elymus kamoji* (Ohwi) S. L. Chen, *Koenigia alpina* (All.) T. M. Schust. & Reveal, *Gentiana algida* Pall., *Rhodiola quadrifida* (Pall.) Fisch. et Mey., *Smelowskia calycina* (Steph.) C. A. Mey.	5–10	0.1–0.3	

## Data Availability

The data presented in this study are available on request from the corresponding author.

## References

[1] The Editorial Committee of Vegetation of China (1983). Vegetation of China.

[2] Kolbek J., Šrůtek M., Box E.O. (2003). Forest Vegetation of Northeast Asia.

[3] Groves C.R., Game E.T., Anderson M.G., Cross M., Enquist C., Ferdana Z., Girvetz E., Gondor A., Hall K.R., Higgins J. (2012). Incorporating climate change into systematic conservation planning. Biodivers. Conserv..

[4] Song Y.C. (2011). Recognition and proposal on the vegetation classification system of China. Chin. J. Plant Ecol..

[5] Cantu C., Wright R.G., Scott J.M., Strand E. (2004). Assessment of current and proposed nature reserves of Mexico based on their capacity to protect geophysical features and biodiversity. Biol. Conserv..

[6] Guo Z.L., Li Z., Cui G.F. (2015). Effectiveness of national nature reserve network in representing natural vegetation in mainland China. Biodivers. Conserv..

[7] Qian Y.B., Zhang H.Y., Wu Z.N., Wang Z.C. (2011). Vegetation composition and distribution on the northern slope of Karlik mountain to Naomaohu basin, east Tianshan mountains. J. Arid Land.

[8] Beket U., Knapp H.D. (2012). Nature conservation and cultural heritage in the Mongolia Altai. Biodivers. Res. Mong..

[9] Cermak J., Opgenoorth L., Miehe G. (2005). Isolated Mountain Forests in Central Asian Deserts: A Case Study from the Govi Altay, Mongolia.

[10] Makunina N.I. (2016). Botanical and geographical characteristics of forest steppe of the Altai-Sayan mountain region. Contemp. Probl. Ecol..

[11] Olonova M.V., Zhang D.Y., Duan S.M., Yin L.K., Pan B.R. (2010). Rare and endangered plant species of the Chinese Altai Mountains. J. Arid Land.

[12] Chen W.L., Yang C.Y. (2000). A floristic study on the seed plant in Mts. Altai China. Acta Bot. Yunnanica.

[13] Pan X.L. (1999). Floristic analysis of seed plant genera in Xinjiang. Bull. Bot. Res..

[14] Chen X.Y., Yan S. (1989). A vegetational survey of valley forests in the pediment plant of Altai district of Xinjiang. Acta Phytoecol. Et Geobot. Sin..

[15] Yang Z.P., Zhang X.L. (1988). Scientific Research on the Kanas Natural Heritage.

[16] Yuan G.Y. (1993). Regional differences and economic significance of vertical natural belts in mountainous regions of Xinjiang. Environ. Prot. Xinjiang.

[17] Liao C., Morreale S.J., Kassam K.A.S., Sullivan P.J., Fei D. (2014). Following the Green: Coupled pastoral migration and vegetation dynamics in the Altay and Tianshan Mountains of Xinjiang, China. Appl. Geogr..

[18] Shayila S., Jiao S.Y. (2008). The measures of grassland ecological protection and construction in Aletai region, Xinjiang. Pratacultural Sci..

[19] Yao F.L., Wang X.C.H., Wang X.T., Yang H.J. (2023). Study on the surface pollen of western Altay Mountains, China. Ecol. Sci..

[20] Zhang D.L. (2021). Variation characteristics of sporopollen type diversity in arid Central Asia under the Holocene westerly mode: A case study of the Altai Mountains. Arid Zone Res..

[21] Li Y.Y., Zhang Y., Kong Z.C., Yang Z.J. (2021). Surface sporopollen and modern vegetation in Hongshanzui area, Altai, Xinjiang, China. Acta Phytoecol. Sin..

[22] Xue R.H., Jiao L., Liu X.P., Chen K. (2021). Evaluation of the stability of the radial growth of *Larix sibirica* at different altitudes in response to climate changein Altai Mountains, Xinjiang. Chin. J. Ecol..

[23] Kang J., Jiang S.W., Huang J.G. (2020). Radial growth response of four dominant tree species to climate factors in the Sayan Range of the Altai Mountains, Russia. Acta Ecol. Sin..

[24] Yeernaer H., Xu X., Dilinuer T., Li H. (2019). Response of Vegetation Coverage to Climate Change in Altai Mountain Forest and Grassland Ecological Function Area in Xinjiang, China. J. Ecol. Rural Environ..

[25] Xinjiang Altay Mountain Forestry Bureau (2004). Two-Riversource Comprehensive Scientific Investigation of the Altay Mountains in Xinjiang.

[26] Feng M., Ren M.L. (1990). Scientific Investigation of Hanasse in Xinjiang.

[27] Feng Y., Zhang Y., Wang X. (2014). Comparison on composition and flora of shrubs between north and south of Xinjiang. J. Plant Resour. Environ..

[28] Chytrý M., Danihelka J., Kubešová S., Lustyk P., Ermakov N., Hájek M., Hájková P., Kočí M., Otýpková Z., Roleček J. (2008). Diversity of forest vegetation across a strong gradient of climatic continentality: Western Sayan Mountains, southern Siberia. Plant Ecol..

[29] Krestov P.V., Ermakov N.B., Osipov S.V., Nakamura Y. (2009). Classification and phytogeography of Larch forests of northeast Asia. Folia Geobot..

[30] Song Y.C. (2001). Ecology of Vegetation.

[31] Yao J. (2008). The Study of RS&GIS-Based Forest Landscape Classification and Distribution Pattern in the Three Gorges Reservoir Area. Master’s Thesis.

[32] Jing X.H. (2008). Study on Landscape Pattern of Vegetation and Spatial Variation of Biodiversitv in the Irtvsh River Basin, Xinjiang. Ph.D. Thesis.

[33] Xinjiang Complex Expert Team of the Chinese Academy of Sciences (1978). The Vegetation and Its Utilization in Xinjiang.

[34] Wang W., Pei H., Wang X. (2016). A logistic analysis on vegetation classification system based on dominant species with an illustrational scheme. Biodivers. Sci..

[35] Chytrý M., Ermakov N., Danihelka J., Hajek M., Hájková P., Horsák M., Kočí M., Kubešová S., Lustyk P., Otýpková Z. (2012). High species richness in hemiboreal forests of the northern Russian Altai, southern Siberia. J. Veg. Sci..

[36] Walter H. (1979). Vegetation of the Earth and Ecological Systems of the Geo-Biosphere.

[37] Zhang X.S. (2007). Vegetation Map of China and Its Geographic Pattern.

[38] Feng Z., Sun A., Abdusalih N., Ran M., Kurban A., Lan B., Zhang D., Yang Y. (2016). Vegetation changes and associated climatic changes in the southern Altai Mountains within China during the Holocene. Holocene.

[39] Schlütz F., Dulamsuren C., Wieckowska M., Mühlenberg M., Hauck M. (2008). Late Holocene vegetation history suggests natural origin of steppes in the northern Mongolian mountain taiga. Palaeogeogr. Palaeoclimatol. Palaeoecol..

[40] Rudaya N., Tarasov P. Mechanisms driving the Holocene vegetation and climate dynamics in central Asia: Case study—The Altai Mountains. Proceedings of the Egu General Assembly Conference.

[41] Tchebakova N.M., Blyakharchuk T.A., Parfenova E.I. (2009). Reconstruction and prediction of climate and vegetation change in the Holocene in the Altai–Sayan mountains, Central Asia. Environ. Res. Lett..

[42] Liu B. (2017). Vertical patterns in plant diversity and their relations with environmental factors on the southern slope of the Tianshan Mountains (middle section) in Xinjiang (China). J. Mt. Sci..

[43] Quintana C., Girardello M., Barfod A.S., Balslev H. (2017). Diversity patterns, environmental drivers and changes in vegetation composition in dry inter-Andean valleys. J. Plant Ecol..

[44] German D., Neuffer B., Friesen N., Hurka H. (2003). Contribution to the knowledge of the Flora of the Mongolian Altai II. Feddes Repert..

[45] Ermakov N., Morozova O. (2011). Syntaxonomical survey of boreal oligotrophic pine forests in northern Europe and Western Siberia. Appl. Veg. Sci..

[46] Neuffer B., Oyuntseg B., Schamsran Z. (2003). Contribution to the knowledge of the Flora of the Mongolian Altai. Feddes Repert..

[47] Polyakova M.A., Dembicz I., Becker T., Becker U., Demina O.N., Ermakov N., Filibeck G., Guarino R., Janišová M., Jaunatre R. (2016). Scale- and taxon-dependent patterns of plant diversity in steppes of Khakassia, South Siberia (Russia). Biodivers. Conserv..

[48] Beket U. (2009). The Vegetation of Mongolia Altai. Problems of Sustainable Land Use and Nature Conservation.

